# N-acetyl-cysteine, a drug that enhances the endogenous activation of group-II metabotropic glutamate receptors, inhibits nociceptive transmission in humans

**DOI:** 10.1186/s12990-015-0009-2

**Published:** 2015-03-20

**Authors:** Andrea Truini, Serena Piroso, Erica Pasquale, Serena Notartomaso, Giulia Di Stefano, Roberta Lattanzi, Giuseppe Battaglia, Ferdinando Nicoletti, Giorgio Cruccu

**Affiliations:** Department of Neurology and Psychiatry, University Sapienza, Rome, Italy; Department of Neurology and Psychiatry, University Sapienza, I.R.C.C.S. Neuromed, Pozzilli, Italy; Department of Physiology and Pharmacology, University Sapienza, Piazzale Aldo Moro, Rome, 500185 Italy; I.N.M. Neuromed, Pozzilli, Italy; Department of Molecular Pathology, I.R.C.C.S. Neuromed, Pozzilli, Italy

**Keywords:** N-Acetylcystene, Neuropathic pain, mGlu2 receptor, Laser evoked potentials

## Abstract

**Background:**

Emerging research seeking novel analgesic drugs focuses on agents targeting group-II metabotropic glutamate receptors (mGlu2 and mGlu3 receptors). N-Acetylcysteine (NAC) enhances the endogenous activation of mGlu2/3 receptors by activating the glial glutamate:cystine membrane exchanger. Here, we examined whether NAC inhibits nociceptive responses in humans and animals. We tested the effect of oral NAC (1.2 g) on thermal-pain thresholds and laser-evoked potentials in 10 healthy volunteers, according to a crossover, double-blind, placebo-controlled design, and the effect of NAC (100 mg/kg, i.p.) on the tail-flick response evoked by radiant heat stimulation in mice.

**Results:**

In healthy subjects, NAC treatment left thermal-pain thresholds unchanged, but significantly reduced pain ratings to laser stimuli and amplitudes of laser-evoked potentials. NAC induced significantly greater changes in these measures than placebo. In the tail-flick test, NAC strongly reduced the nocifensive reflex response to radiant heat. The action of NAC was abolished by the preferential mGlu2/3 receptor antagonist, LY341495 (1 mg/kg, i.p.).

**Conclusions:**

Our findings show for the first time that NAC inhibits nociceptive transmission in humans, and does the same in mice by activating mGlu2/3 receptors. These data lay the groundwork for investigating the therapeutic potential of NAC in patients with chronic pain.

## Background

Despite recent advances, only few patients suffering from chronic pain achieve acceptable pain relief with the currently available analgesic drugs [1].

Potential targets for novel analgesic drugs include group-II metabotropic glutamate receptor subtypes (mGlu2 and mGlu3 receptors) [2,3]. mGlu2 and mGlu3 receptors localized in the spinal cord and other regions of the nociceptive system negatively regulate glutamate release by reducing cAMP formation and inhibiting voltage-sensitive calcium channels in presynaptic terminals [2,4]. Immune gold labelling has shown that both receptors are preferentially (albeit not exclusively) localized in the preterminal axonal region, and are therefore inaccessible to synaptic glutamate. A growing body of evidence suggests that presynaptic mGlu2 and mGlu3 receptors are activated by the glutamate released from astrocytes *via* the glutamate:cystine antiporter (Sxc-) [5,6]. A drug that activates Sxc-, and might therefore be used to reinforce the endogenous activation of mGlu2/3 receptors, is N-acetylcysteine (NAC) [7]. We have shown recently that NAC induces analgesia in animal models of inflammatory and neuropathic pain [8]. NAC-induced analgesia was abolished by genetic deletion of mGlu2 receptors or by co-treatment with the preferential mGlu2/3 receptor antagonist, LY341495 [8]. The study of nociceptive transmission in healthy volunteers is an essential step towards the clinical research of NAC in patients with chronic pain.

In this study we examined whether oral NAC was able to modulate nociceptive transmission in healthy volunteers. Using a double-blind, placebo-controlled design, we tested NAC-induced changes in quantitative sensory testing and laser-evoked potentials, two techniques that international guidelines indicate as “reference standards” for assessing the nociceptive system and testing analgesic efficacy [9-11]. As an experimental counterpart in animals, we also examined changes induced by intraperitoneal-injected NAC on the tail-flick test elicited by radiant heat in mice, the animal model most closely corresponding to laser stimulation in humans.

## Results

### NAC-induced changes in nociceptive transmission in humans

No subjects reported adverse events after receiving NAC or placebo. The NAC and placebo sessions yielded comparable baseline values for quantitative sensory testing and laser-evoked potential variables (P > 0.1; Table 1). Baseline values were within the normal ranges established in our laboratory.

**Table 1:** **Effect of oral NAC (1.2 g) and placebo on nociceptive transmission in ten healthy volunteers**

	**Placebo**			**NAC**			**Placebo**	**NAC**	
	**Pre-drug**	**Post-drug**	**P**	**Pre-drug**	**Post-drug**	**P**	**Changes Pre-Post**	**Changes Pre-Post**	**P**
N1-LEP (μV)	7.1 ± 0.8	8.5 ± 0.6	NS	8.3 ± 1.4	4.8 ± 0.8	<0.02	1.6 ± 0.8	- 3.6 ± 3.8	<0.02
N2P2-LEP (μV)	30.9 ± 2.1	29.3 ± 2.1	NS	35.6 ± 3.7	21.8 ± 3.1	<0.01	-1.6 ± 1.9	-13.9 ± 3.9	<0.01
Laser pain (NRS 0-10)	5.6 ± 0.2	5.5 ± 0.2	NS	6.3 ±0.5	5.3 ± 0.5	<0.01	0.05 ± 0.6	-1.0 ± 0.2	<0.01
Cold detection threshold (°C)	29.1 ± 0.4	28.7 ± 0.5	NS	28.8 ± 0.4	28.7 ± 0.6	NS	-0.4 ± 0.4	-0.1 ± 0.5	NS
Warm detection threshold (°C)	34.8 ±0.5	35.9 ± 0.8	NS	34.2 ± 0.2	35.0 ± 0.4	NS	1.1 ± 0.8	0.8 ± 0.3	NS
Cold pain threshold (°C)	13.8 ± 2.4	13.6 ± 2.3	NS	13.4 ± 2.5	14.8 ± 2.5	NS	-0.2 ± 1.7	1.4 ± 1.7	NS
Heat pain threshold (°C)	43.5 ± 0.7	43.7 ± 0.7	NS	43.8 ± 1.1	43.7 ± 1.1	NS	0.2 ± 0.5	-0.1 ± 0.5	NS

Values are means ± S.E.M.

**Figure 1:**
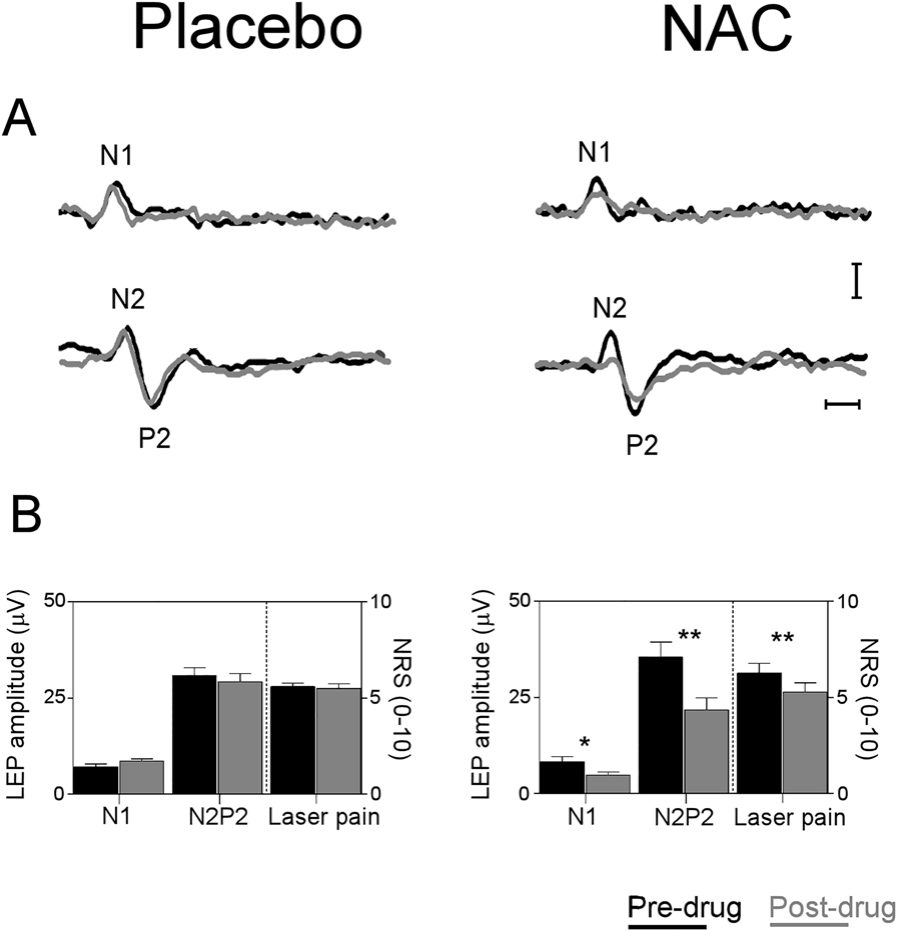
**Oral NAC inhibits nociceptive transmission in healthy volunteers. A**: Laser-evoked potential (LEP) recordings during placebo and N-acetyl-cysteine (NAC) sessions in a representative subject. Black: pre-drug recordings. Grey: post-drug recordings. Each trace is the mean of 20 trials. Horizontal calibration: 100 ms; vertical calibration: 20 μV. **B**: Mean pre-drug (black) and post-drug (grey) values for N1, N2-P2 amplitude LEP components and laser pain ratings during placebo and NAC sessions. Whereas placebo was ineffective, NAC significantly reduced all LEP components and laser pain ratings. Values are means + S.E.M. of 10 determinations. *p < 0.02; **p < 0.01.

Oral NAC (1.2 g) significantly attenuated pain evoked by laser stimuli, and the N1-and N2P2 laser-evoked potential amplitudes (laser pain ratings: p < 0.01; N1 component: p < 0.02; N2-P2 complex: p < 0.01) (Figure 1), but left thermal and pain perceptive thresholds unchanged. Placebo left all variables unchanged (Table 1).

### NAC-induced changes in tail-flick latencies in mice

**Figure 2:**
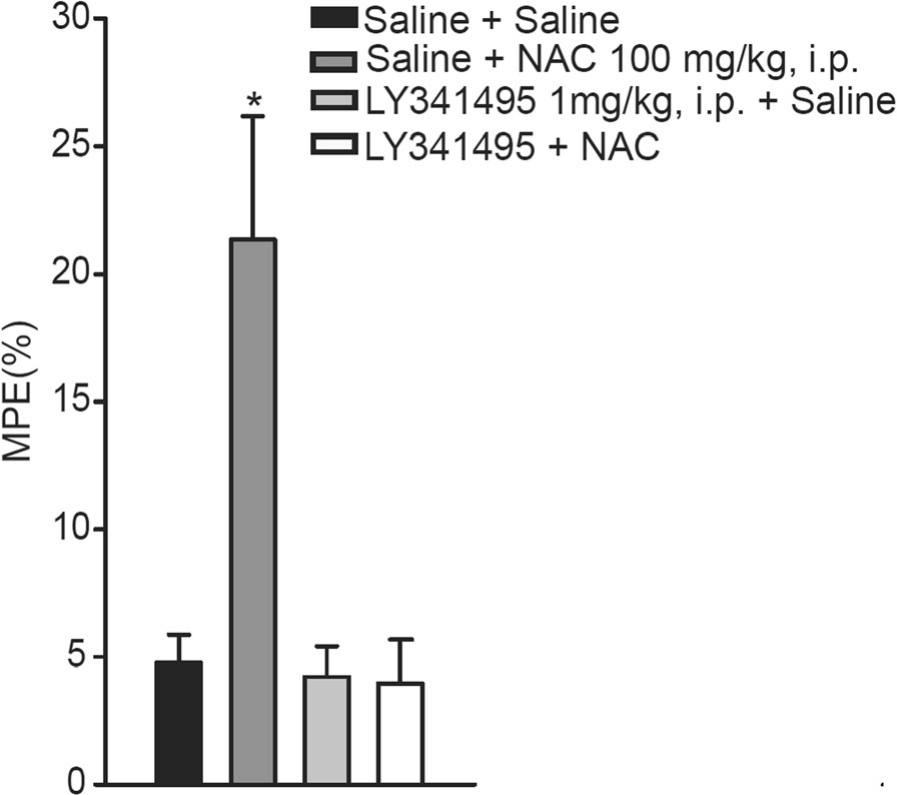
**Acute injection of NAC inhibits radiant heat-induced nocifensive behavior in mice by activating mGlu2/3 receptors.** Percentage of the maximum possible effect (%MPE) in the four tail-flick conditions: **i)** saline followed by saline; **(ii)** saline followed by N-acetyl-cysteine (NAC) (100 mg/kg); **(iii)** LY341495 (1 mg/kg) followed by saline; and **(iv)** LY341495 followed by NAC. NAC injection increased tail-flick latencies. Values are means + S.E.M. of 6–8 determinations. *p < 0.05 vs. all other values, F_(3,22)_=6.38, p=0.003.

A single injection of NAC (100 mg/kg, i.p.; 30 min before the test) substantially increased tail-flick latencies as compared with saline injection in mice (p=0.018) (Figure 2). Pretreatment for 15 min with the preferential mGlu2/3 receptor antagonist, LY341495 (1 mg/kg, i.p.), which was inactive on its own, abolished NAC-induced analgesia (p=0.017) (Figure 2).

## Discussion

Our study, showing that oral NAC reduced laser pain ratings and laser-evoked potential amplitudes, provides the previously unavailable evidence that NAC inhibits nociceptive transmission in healthy humans. These findings in humans received confirmation from the NAC-induced tail-flick suppression in mice.

Precisely where in the nervous system NAC acts to induce analgesia in the nociceptive transmission pathway remains unclear. Although mGlu2/3 receptors lie at various levels across the nociceptive pathway [4], we hypothesize that it does so by inhibiting neurotransmitter release at the synapse between first-order and second-order nociceptive neurons in the dorsal horn [12]. This hypothesis is consistent with our data of laser-evoked potentials showing that NAC concomitantly reduced the N1 component (generated in the SII area), and the N2-P2 complex (generated in the insular and cingulate cortex) [13], which is indicative of a lower action of the drug within the nociceptive pathway. The hypothesis that NAC induces its analgesic effect predominantly in the dorsal horn is also supported by data of the formalin test in mice. NAC specifically reduced nocifensive behavior in the second phase of the formalin test [8], which reflects the development of nociceptive sensitization in the dorsal horn of the spinal cord [14].

Whereas NAC reduced the laser pain ratings and laser-evoked potential amplitudes, it left the thermal-pain thresholds-assessed by the quantitative sensory testing-unchanged. This finding is not surprising because analgesic agents, such as opioids and monoaminergic drugs, have more pronounced effects on high-intensity pain (e.g., laser-evoked pain) than on thermal-pain thresholds [11,15,16].

Unexpectedly in our study we did not find any placebo effect on laser-evoked potentials. This finding is not in line with some studies demonstrating that placebo affects laser-evoked potentials [17]. There are the following potential explanations: (i) in our experiments the subjects received information on the aim of the study; (ii) our subjects knew they had the chance to receive placebo; and (iii) all subjects were well-trained neurophysiologists and neurologists of our Department, and this might have minimized the placebo effect.

As expected in none of our subjects did NAC cause adverse events. NAC, commonly used as a mucolytic agent, is an extremely safe drug. The standard dose (600 mg/day) induces negligible adverse effects [18]. Even when given at high I.V. doses in the treatment of acetaminophen poisoning, its adverse effects are usually mild and easily managed [19]. Hence, NAC might be an ideal and safe adjuvant drug for treating patients with chronic pain undergoing multiple treatments for pain and the related comorbidities.

Using a radiant heat experimental pain model--similar to that used in humans--our experiments showed that tail-flick responses had a longer latency in NAC-treated mice than in saline-treated mice. The tail-flick delay was sensitive to the LY341495-induced mGlu2/3 receptor block. These findings are consistent with the analgesic effect of NAC reported in other experimental pain models (e.g. the formalin test, the complete Freund’s adjuvant chronic inflammatory pain model, and in the chronic constriction injury neuropathic pain model) [8], and show that mGlu2/3 receptors mediate the NAC-induced changes in nociceptive transmission in mice.

## Conclusions

Our study, showing that NAC reduces laser-induced pain and laser-evoked brain potentials in humans and delays the tail-flick response in mice indicates that NAC inhibits nociceptive transmission. Accordingly, a previous study directly addressing the efficacy of NAC in patients with complex regional pain syndrome type I, has raised the possibility that NAC might be potentially useful for relieving pain [20]. Hence, because NAC is a safe drug, it deserves to be tested in further adequately sized clinical trials in patients with pain.

## Methods

### Human experiments

Ten healthy volunteers (4 M, 6 F; 23–38 years) participated in the study. The Institutional Review Board approved all procedures and all subjects gave their written informed consent.

The Institutional Review Board of the Policlinico Umberto I – Department of Neurology and Psychiatry, Sapienza University approved all procedures and all subjects gave their written informed consent.

### Quantitative sensory testing of thermal-pain sensitivity

For quantitative sensory testing we used a thermode (ATS, PATHWAY, Medoc, Israel). The computer-driven PATHWAY system contains a metal contact plate (contact area 30 × 30 mm) equipped with an external Peltier element that cools and heats the plate to target levels. The baseline temperature of 32°C reached target temperature at a ramp rate of 1°C/s. Quantitative sensory variables were tested on the right hand dorsum. We tested subjects’ thermal-pain perceptive thresholds: cold detection threshold (CDT), warm detection threshold (WDT), cold pain threshold (CPT) and heat pain threshold (HPT).

### Laser-evoked potentials

We used a Nd: YAP laser stimulator under fiber–optic guidance (Electronic Engineering, Florence, Italy). Laser stimuli were set to induce a clear painful pinprick (intensity 119.4-150 mJ/mm^2^; duration 5 ms; diameter 4 mm) and directed to the right hand dorsum. The laser beam was shifted after each stimulus and the interstimulus interval was varied pseudo-randomly (10–15 s). Subjects, wearing protective goggles, rested comfortably on a medical examination table, keeping their eyes open. The different laser evoked potential components were recorded through disk electrodes from the scalp: T3 referenced to Fz for recording the early lateralized N1 component, and Cz referenced to the nose, for recording the late vertex N2-P2 complex. Electro-oculographic recordings monitored possible eye movements or blinks. For each session, two series of 10–15 artefact-free trials were averaged off line. We measured the peak latencies and amplitudes of the lateralized N1 and the vertex N2-P2 complex. These methods adhered to the recommendations given by the International Federation of Clinical Neurophysiology [21]. In all sessions the subjects were asked to rate the pain evoked by laser stimuli on a 0–10 numeric rating scale (NRS) (0=no sensation, 10=worst possible pain).

### Experimental procedure

We conducted a double-blind, placebo-controlled, crossover study. All subjects underwent two separate sessions, one with oral N-acetylcysteine (NAC) (effervescent tablets, Ratiopharm, 1.2 g) and the other with oral placebo (Multicentrum, multivitamin supplement, in effervescent tablets). The two sessions were randomly alternated among subjects. Two investigators, both unaware of the type of session (drug or placebo), recorded the thermal-pain perceptive thresholds and laser evoked potential measures. Each session comprised two recording blocks: before oral NAC or placebo (pre-drug), and 60 min after NAC or placebo (post-drug). As primary outcome variables, we selected the warm and cold detection thresholds, the cold and heat pain thresholds; N1 component and N2-P2 laser-evoked potential amplitudes and the numerical rating scale for the perceived pain intensity during the laser evoked potential recordings.

### Animal experiments

All experiments were conducted according to the European (86/609/EEC) and Italian (D: Lgs. 116/92) guidelines for animal care. The experimental protocol was approved by the Italian Ministry of Health. All efforts were made to minimize animal suffering and the number of animals used.

### Drugs

NAC was purchased from Sigma Aldrich (Milano, Italy). (2S)-2-Amino-2-[(1S,2S)-2-carboxycycloprop-1-yl]-3-(xanth-9-yl) propanoic acid (LY341495) was purchased from Tocris Cookson (Avonmouth, Bristol, UK).

### Tail-flick test

We used adult male C57BL/6 J mice (body weight 25–28 g) purchased from Charles River (Calco, Italy). All mice were housed 5 per cage, under a standard 12/12 h light/dark cycle with food and water *ad libitum*. Testing took place from 9:00 a.m. to 11 a.m. on two consecutive days. Four groups of 6–8 mice received two intraperitoneal (i.p.) injections separated by a 15-min interval, as follows: (i) saline followed by saline; (ii) saline followed by NAC (100 mg/kg); (iii) LY341495 (1 mg/kg) followed by saline; and (iv) LY341495 followed by NAC. In the tail-flick test, mice were loosely wrapped in a velvet towel and placed on the tail-flick apparatus (Tail Flick model DS 20 Socrel Apelex, France). A light beam was focused on the tail approximately 1 to 3 cm from the base, and to minimize tissue damage the latency to a vigorous radiant-heat-induced tail flick was measured with a 10-second cut-off time. Tail-flick latencies at baseline (BL) were measured 3 times per mouse at 2-min intervals, and 5 min before the first i.p. injection. The study included only mice whose mean baseline reaction times ranged between 3 and 5 sec. Latencies were measured again 30 min after the second i.p. injection (test latency, TL). Results were expressed as a percentage of the maximum possible effect (%MPE) using the following formula: %MPE=100_{(TL–mean BL)/(Cut-off time–mean BL)}.

### Statistical analysis

Because several variables for human subjects had a non-normal distribution, all comparisons in humans were analyzed for significance with the Wilcoxon signed-rank test. Data for tail-flick in mice were tested with a one-way analysis of variance (ANOVA) followed by Dunnett’s *t* test to isolate the differences. P values <0.05 were considered as statistically significant.

## Abbreviations

mGlu: Metabotropic glutamate receptor
Sxc-: Cystine/glutamate antiporter
NAC: N-Acetylcysteine
CDT: Cold detection threshold
WDT: Warm detection threshold
CPT: Cold pain threshold
HPT: Heat pain threshold
